# Methodology development for analyzing eating behavior, diet quality, and food environment in occupational settings

**DOI:** 10.1177/20552076261467799

**Published:** 2026-07-16

**Authors:** Valeria Cecchini, Negar Zand Miralvand, Vasileios Papapanagiotou, Carla Maria Avesani, Ioannis Ioakeimidis

**Affiliations:** 1 Renal Research Novum, Division of Renal Medicine, Department of Clinical Science, Intervention and Technology, 27106 Karolinska Institutet, Stockholm, Sweden; 2 Innovative Use of Mobile Phones to Promote Physical Activity and Nutrition Across the Lifespan (the IMPACT) Research Group, Department of Medicine (MedH), 27106 Karolinska Institutet, Stockholm, Sweden

**Keywords:** digital health, mobile application, occupational health, eating behavior, food environment, ultra-processed foods

## Abstract

**Background:**

Mobile-based dietary assessment tools have the potential to capture real-time eating behaviors and food environment exposure, but their feasibility and usability in workplace contexts remain underexplored.

**Objective:**

To evaluate the feasibility and usability of the Mobile Sense App among employees and students at the Karolinska Institute.

**Methods:**

This study employed the Mobile Sense App, which was developed to record dietary intake and food purchasing patterns through photographs and geospatial data. The study combined data from the pre-pilot and pilot studies (n=56). Participants, who were students and employees at a public institution, completed a baseline questionnaire and used the application (app) for two weeks to photograph all meals, snacks, and drinks consumed during working hours on campus. Adherence was evaluated based on the number of photographs submitted over a six-day monitoring period. Usability was assessed using the System Usability Scale (SUS). Image data were manually annotated, ultra-processed foods (UPFs) were identified according to the NOVA classification system, and inter-rater reliability was evaluated using Cohen’s κ. Food purchasing ‘hotspots’ were examined through geospatial analyses.

**Results:**

Participants submitted n=1178 photographs. Cohen’s κ indicated strong agreement between independent researcher annotation (κ=1.00 for meals/snacks; κ=0.91 for drinks). The mean SUS score was 75.2 ± 12.2. Store-bought meals showed a higher prevalence of UPFs (64%) than home-made meals (13%) (χ^2^ (1)=178.63, p<.001). Global Positioning System (GPS) data indicated food purchases clustered around campus dining areas. Open-ended feedback emphasized the need for reminders, clarity in user interface (UI) instructions, and flexibility in annotation options as desired improvements.

**Conclusions:**

The proposed approach is a feasible and scalable methodology for real-world dietary monitoring, supporting further evaluation for larger-scale implementation.

## Introduction

Poor diet quality remains a major public health concern worldwide, contributing to non-communicable diseases (NCDs) and reduced quality of life.^1^ These diet-related illnesses translate into substantial societal costs, including increased healthcare expenditures and productivity losses, mainly through absenteeism.^2^ In Sweden, obesity is associated with lifetime productivity losses estimated at € 95 000 per person, nearly twice that of normal-weight counterparts.^3,4^ A significant contributor to poor diet quality is the growing consumption of ultra-processed foods (UPFs). High UPF intake has been linked to a wide range of adverse health outcomes, including cardiometabolic diseases,^5,6^ cancer,^7^ kidney disease,^8^ and all-cause mortality.^7^ These risks are amplified by their high availability and widespread marketing.^9^

According to the NOVA classification system, UPFs are ‘formulations of ingredients, mostly of exclusive industrial use, that result from a series of industrial processes’, typically containing little or no whole foods and often including food additives, such as flavor enhancers, colorants, emulsifiers, and preservatives to restore palatability and increase shelf-life.^9^ Nutritionally, these products are usually high in free sugars, saturated fats, energy density, and have a multitude of ingredients, including cosmetic additives, while lacking fiber, protein, micronutrients, and bioactive compounds.^9,10^

The workplace food environment plays a critical role in shaping UPF consumption.^11^ The availability of UPFs in schools, universities, and workplaces affects dietary habits, influencing both the decision to consume UPFs and the frequency of consumption.^12^ Among these environments, occupational settings are important, as adults spend a large portion of their waking hours at work.^13^ Worksite factors such as food availability, time constraints, social norms, and organizational culture further shape dietary patterns in ways that may increase UPF consumption and decrease overall diet quality.^14^ Thus, understanding eating behaviors and the surrounding food environment are essential for both public health research and workplace health promotion.

Despite its importance, collecting reliable dietary data in real-world contexts remains challenging. Conventional dietary assessment methods, such as 24-hour recalls, food frequency questionnaires, and food diaries are usable tools, but remain burdensome for participants, prone to recall bias, and often yield incomplete or imprecise data.^15^ Digital health technologies, particularly mobile applications (apps), offer promising solutions to these challenges. In this context, smartphones can enable real-time, *in situ* dietary data collection, reducing recall error, lowering participant burden, and enhancing ecological validity.^16^ Overall, these methodologies can provide valuable tools for investigating dietary habits in relation to the food environment in which they occur. By incorporating features such as time-stamped photographs and geolocation data, mobile tools can provide a richer context for dietary assessment than traditional methods. Yet, despite this potential, methodological frameworks for evaluating the feasibility and usability of such tools in occupational settings remain underdeveloped. Feasibility and usability are often reported as secondary outcomes, leaving a gap in guidance on how to systematically design, implement, and evaluate mobile dietary assessment methodologies in workplace contexts.^17^ While photographic logging has been previously used and proven effective for capturing detailed dietary intake,^18,19^ spatial data derived from geolocation is less frequently employed, despite its potential to map food environment exposures and workplace-specific eating patterns. Integrating both photographic and spatial analytics can provide richer insights into not only what employees consume, but also in which environment. This combined methodology offers a more complete understanding of workplace food behaviors and the contextual drivers of dietary quality.

Taking these challenges into consideration, the current study was designed to develop and evaluate a methodological framework for capturing dietary behaviors and food environment exposures in an occupational context, specifically a university setting. This study represents the first deployment in Sweden of a dedicated digital dietary assessment and food environment data collection and reporting system, integrating data capture through the Mobile Sense App (Arbisense AB, Stockholm, Sweden) with centralized portal analytics.

The primary aim of this pilot study was to test the feasibility and usability of the Mobile Sense App for capturing eating behavior and food environment exposure in a real-world university setting, in order to assess its technical validity, user acceptance, and scalability. This approach enabled differentiation between home-made and store-bought meals, while simultaneously mapping purchase locations across the campus environment to identify UPF prevalence and purchasing ‘hotspots’, respectively.

## Material and methods

### Study setting and participants

This study was conducted in three buildings located at the southern campus of the Karolinska Institute (KI), Stockholm, Sweden. Participants included both employees and students working or studying within these facilities. No exclusion criteria were applied for participating in the study; however, only participants that registered at least three meals per week on campus had their data included in the analysis. The study followed an observational, cross-sectional design. Data were collected over two main phases, each lasting two weeks: a pre-pilot phase in February 2025 and a pilot phase in May 2025. A subset of participants (n=6) took part in both the pre-pilot and pilot phases of the study. To avoid duplicate reporting and provide a comprehensive overview of app usage and dietary data, results from the two phases were merged. For participants who contributed with baseline questionnaire data in both phases, only their first set of submissions was included in the combined dataset.

### Recruitment and consent

Recruitment was conducted using a convenience sampling approach, consistent with the feasibility and methodology-development focus of the study. Participants were recruited through multiple channels. During the study’s pre-pilot phase, researchers informed colleagues within their immediate professional networks about the study, and interested individuals received a personalized weblink granting access first to the digital informed consent and then to a fully anonymized baseline questionnaire. Both were administered via REDCap (Research Electronic Data Capture) hosted at KI. REDCap is a secure, web-based software platform designed to support data capture for research studies.^20,21^

**Figure 1.**
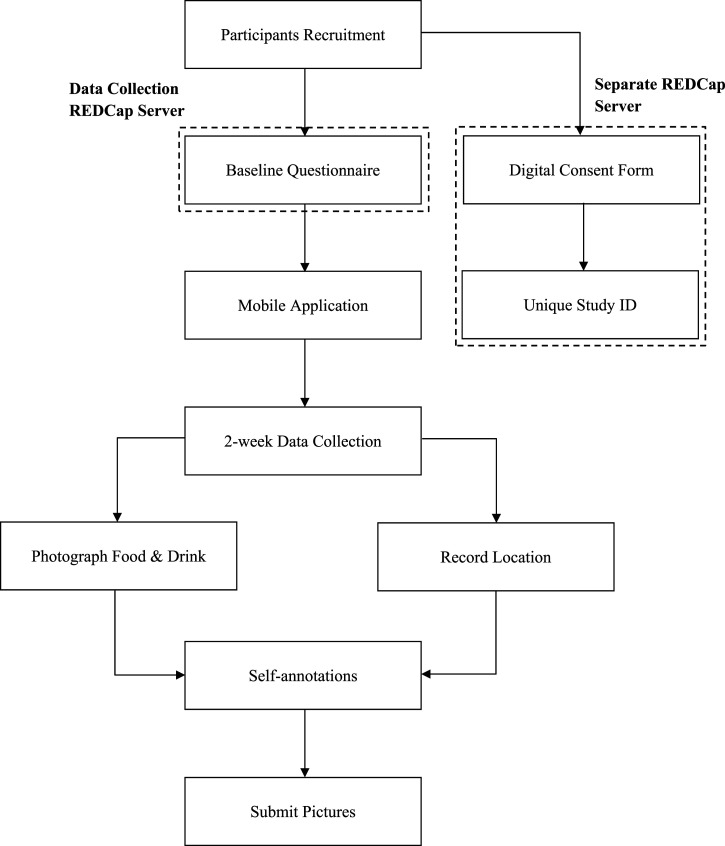
Study recruitment and data collection procedure during the pre-pilot and pilot phases of the study.

The study recruitment and data collection procedure, presented in Figure 1, began with a digital consent form, available in both English and Swedish, which participants completed through a password-protected link sent by email. After signing digitally, each participant was assigned a unique study ID. Subsequently, they received a personalized link to a baseline questionnaire, which collected descriptive and lifestyle information, including age, sex, student/employee status, body weight, height, work hours, eating habits, physical activity, sleep quality, and stress. Height and weight data were used to calculate body mass index (BMI). Notably, the baseline questionnaire also captured participants’ on-campus eating behaviors, including the frequency of food consumption and purchases, as well as the type of meals, snacks, and beverages they obtained within the campus area. These responses were stored on a separate KI REDCap server, ensuring that no identifiable information could be traced back to participants. Specifically, the photograph submissions from participants were fully anonymized. This approach safeguarded participants’ privacy and allowed analyses to be conducted at a population level without exposing individual information.

In the study’s pilot phase, recruitment was further supported by digital announcements posted on the institution’s internal communication channels, as well as physical notices displayed near dining areas and building entrances. In addition, members of the research team visited lecture halls in person to inform the students about the study.

### Mobile Sense App and geo-environmental portal

The Mobile Sense App is a research tool that integrates real-time food logging via smartphone photographs with geolocation and timestamp data, all of which are linked to a secure portal for aggregation and visualization. Each photograph captured with the app contained anonymized Global Positioning System (GPS) data, allowing the development of maps of frequently visited consumption and purchase sites, hereafter referred to as “hotspots”.

**Figure 2.**
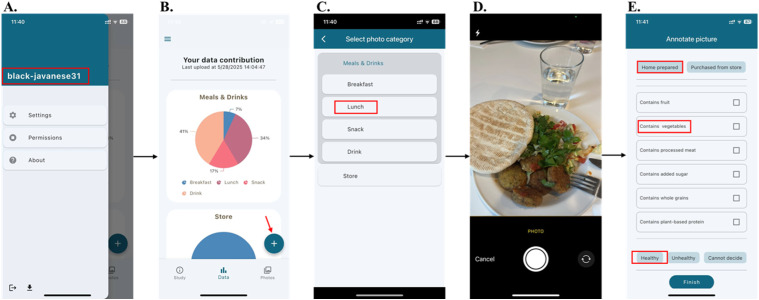
Photograph uploading process in the Mobile Sense App. Panel A displays an anonymized participant ID, Panel B shows usage statistics for the contributor, and Panels C–E illustrate the photograph submission workflow, from selecting the meal category (c), capturing the image (d), to annotating it within the app (e).

To support sustained engagement, participants received daily lunchtime reminders to upload photographs. They also had access to daily statistics of their app usage (e.g., number of photos submitted, proportion of meals logged) and an overview of their own food-related photographs, as showed in Figure 2(b). Figure 2 illustrates the steps involved in uploading photographs trough the app interface.

The Mobile Sense App has previously been deployed in similar research projects such as *AdSafe*,^22^ *FoodShift pathways*,^23^ *and Digifood*^24^ that focused on group level information targeting children and school environments. In this pilot study, the data collection tool was adapted and evaluated, for the first time, to capture dietary habits among adults, specifically within a university setting.

### Use instructions provided to participants

After completing consent procedures, participants were provided access to the Mobile Sense App and were instructed to use it over a two-week period, submitting up to ten photographs per day. Specifically, they were asked to: 1) photograph all foods and beverages consumed on campus premises and indicate whether these were home-made or purchased; 2) record locations where food was purchased, such as restaurants, outlets, or supermarkets on and around the campus; and 3) annotate their submissions with additional details, such as whether the food contained processed meat or had a salty or sweet taste. These annotations were optional.

### Researcher training and annotation scheme adaptation

Initially, two independent researchers adapted the annotation schemes developed for previous studies for the needs of the current study. The newly developed scheme captured information on the meal source (including home-made, store-bought, restaurant, café, fast food, and supermarket), visible branded products, the presence of UPFs, classified according to the NOVA classification system,^9^ and UPF subcategories. Similarly, drink annotations included source, type of sweeteners, presence of UPFs, and UPF subcategories. To compare UPF prevalences across food sources, meals were grouped into two categories, home-made and store-bought (including all purchased meals). For beverages, the store-bought category included sodas, juices, and coffee or tea obtained from workplace machines, whereas only water and home-made smoothies were classified as home-made.

Subsequently, 600 meal photographs from a standardized training dataset (collected for a separate study in 2024), were independently annotated by two researchers using the schemes developed for the present study. This process was part of the researchers’ training. The inter-rater reliability showed strong agreement, and this step ensured the suitability of the schemes for all food categories depicted in the photographs. Additionally, it facilitated the development of standardized guidelines for food categorization and photograph annotation applicable to future studies.

### Annotations of the main dataset

For the main dataset, all submitted photographs were downloaded from the server and randomized. Photograph annotation was performed using a dedicated image annotation software, with each photograph independently reviewed by two researchers according to the pre-defined annotation schemes developed for meals and drinks.

Geospatial analyses were conducted in a dedicated portal developed by Arbisense AB, which enabled visualization of the spatial distribution of submitted photographs. Anonymized geo-tagged data collected through the mobile app were used to map meal purchase locations and to identify activity “hotspots”. The same platform also provided access to photographs alongside their respective metadata analytics, user self-annotations, and descriptive statistics.

Supplementary Figure 1 illustrates the meal annotation procedure, while Supplementary Figure 2 displays photograph annotation interface and associated metadata in the Arbisense Portal, including category, date, time, and user-provided annotations.

### Feasibility and usability

The app’s feasibility was evaluated by examining participants’ adherence to photograph submissions over a six-day period following their initial submission. For each participant, the first submitted photograph marked the start of monitoring, after which the number of photographs submitted over a six-day period was recorded.

During the pre-pilot phase, participants evaluated the usability of the Mobile Sense App through the System Usability Scale (SUS), app-specific usability questions (‘yes’/‘no’/‘I don’t know’), and open-ended feedback, which offered additional insights into user experience and the data collection process. The open-ended feedback was analyzed using inductive thematic analysis.^25^ Responses were read repeatedly to ensure familiarization with the data, and initial codes were generated to capture key meanings and patterns. These were then grouped into broader themes reflecting *usability*, *functionality*, and *engagement aspects* of the Mobile Sense App. Findings from these assessments informed improvements to the app and study procedures, optimizing the pilot phase.

The SUS score was calculated by converting each response, rated on a five-point Likert scale, into a standardized value: for odd-numbered items, the raw score minus 1 was used, while for even-numbered items, the score was subtracted from 5. This yielded values on a 0-4 scale that were summed across all 10 items, and the total was then multiplied by 2.5 to generate a final SUS score ranging from 0 to 100, where higher scores indicated better usability.^26^

### Statistical analysis

Descriptive statistics, mean ± standard deviation (SD), frequency (n and percentage), were used to summarize demographic and photograph characteristics, and usability. Differences in the proportion of UPFs between store-bought and home-made meals were assessed using the chi-square test with a significance level of p<0.05. Annotation reliability was evaluated by comparing independent annotations of the same dataset. Inter-rater reliability between the two researchers who annotated the photographs was assessed using Cohen’s kappa coefficient (κ) to quantify agreement and consistency in food classification. These were interpreted according to established benchmarks, with values above 0.60 considered substantial and above 0.80 almost perfect agreement.^27^ All statistical analyses were conducted using IBM SPSS Statistics (version 31.0.0.0, Armonk, NY, USA).

### Ethical approval

The current study was approved by the Swedish Ethics Review Authority (DR.nr. 2024-07535-01; date 2024-12-10). Participants provided written digital informed consent before the start of the study.

## Results

The study comprised a total of n=1178 photographs collected from n=62 participants, of which n=6 participated in both phases of the study. Inter-rater reliability was high, with Cohen’s κ coefficients of 1.00 for meal and snacks photographs and 0.91 for drinks, indicating almost perfect agreement between the two annotation datasets.

### Baseline self-reported participant characteristics

Demographic characteristics and weekly working hours are summarized in Table 1. The majority of participants were females (84%), and the largest age group was between 24-34 years (39%). Weekly working hours varied, with most participants reporting 31-40 hours (41%) per week. The mean BMI was 22.5 ± 2.8 kg/m^2^.

**Table 1.** Baseline demographic characteristics of employees and students at the Karolinska Institute South Campus.

Characteristic	Total (n=56)
**Sex**	**n (%)**
Female	47 (84%)
Male	9 (16%)
**Occupation**	
Employee	40 (71%)
Student	16 (29%)
**Age**	
18-24	6 (11%)
24-34	22 (39%)
35-44	16 (29%)
45-54	9 (16%)
Over 54	3 (5%)
**Work hours at the workplace per week**	
Less than 10 hours	2 (4%)
10-20 hours	12 (21%)
21-30 hours	10 (18%)
31-40 hours	23 (41%)
More than 40 hours	9 (16%)
​	**Mean (SD)**
**BMI (kg/m** ^**2**^ **)**	22.5 (2.8)

BMI, body mass index; SD, standard deviation.

### Baseline self-reported food consumption patterns

As shown in Table 2, home-made meals were the predominant food choice among participants, with 41% reporting consumption 4-5 times per week. In contrast, store-bought meals were less common, with 39% indicating consumption about once per week.

**Table 2.** Frequency of home-made and store-bought meals and snack consumption during working hours.

Type of food and frequency of consumption	Total (n=56)
**Home-made meals**	**n (%)**
Once a week	6 (11%)
2-3 times per week	22 (39%)
4-5 times per week	23 (41%)
Never	5 (9%)
**Store-bought meals**	
Once a week	22 (39%)
2-3 times per week	16 (29%)
4-5 times per week	5 (9%)
Never	13 (23%)
**Store-bought snacks**	
Once a week	20 (36%)
2-3 times per week	13 (23%)
4-5 times per week	1 (2%)
Never	22 (39%)

**Figure 3.**
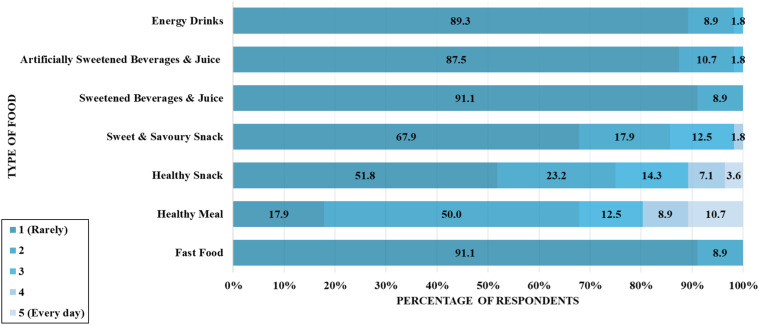
Frequency of consumption of various foods during working hours as reported in the baseline questionnaire. Responses are self-rated on a 1-5 scale (1 = rarely, 5 = every day).

The baseline self-reported consumption frequency of different foods during work hours is shown in Figure 3. Intake of fast foods, sweetened and artificially sweetened beverages, and energy drinks was low, being reported as rarely consumed by more than 85.0% of the total sample. Savory and sweet snacks were also infrequently consumed, as 67.9% reported rare consumption. Healthy meals, in contrast, were more frequently consumed; most of the participants (50.0%) had moderate intake (rating 2). Healthy snacks showed a mixed pattern, with 51.8% consuming them rarely, but a minority (23.2% and 14.3%) consuming them moderately.

### Feasibility and engagement

**Figure 4.**
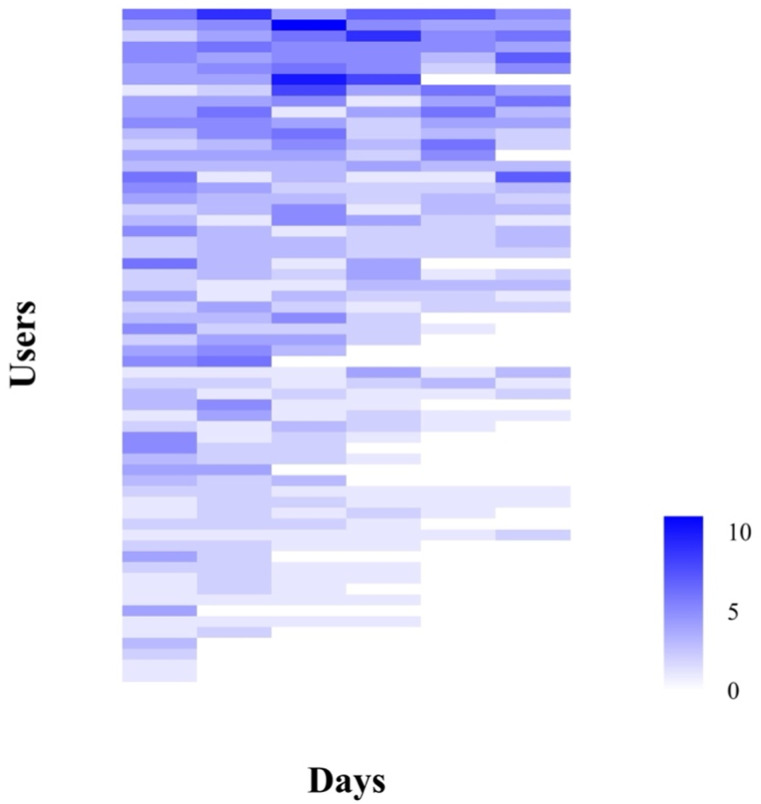
Heatmap of participants’ engagement and photograph submission patterns in the Mobile Sense App over a six-day period. Days with higher color intensity represent higher photograph submission by participants. Participants were ranked by the total number of photographs they submitted, from highest to lowest.

A total of n=62 participants submitted photographs over the two phases of the study. To evaluate user engagement and assess the feasibility of sustained participation, the total number of photographs submitted per participant over a standardized six-day period was analyzed. Figure 4 presents a heatmap of submission activity, illustrating temporal patterns of engagement and highlighting days of higher versus lower interaction with the Mobile Sense App.

### System usability and user feedback during data collection

**Figure 5.**
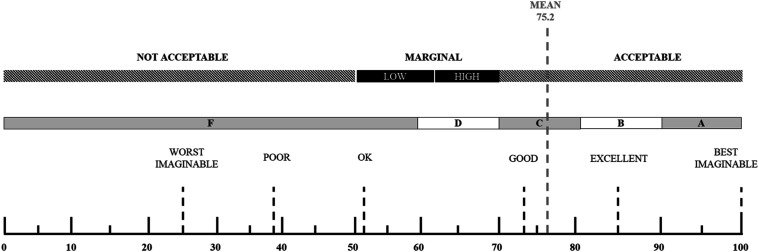
System Usability Scale (SUS) score and acceptability of the Mobile Sense App. The dotted line represents the app’s mean SUS score calculated for the current study.

Figure 5 presents the results of the SUS score for the Mobile Sense App, which achieved a mean score of 75.2 ± 12.2. Table 3 shows participants’ responses (‘yes’/‘no’/‘I don’t know’) to the app-specific usability questions.

**Table 3.** App-specific usability questions.

​	Questions	Total (n=23)		
		Yes	No	I don’t know
**1**	Based on your experience as a participant, would you mind sharing the same type of data, in other contexts in the future?	6 (26.1%)	17 (73.9%)	0 (0%)
**2**	Do you feel insecure about the confidentiality of your data while using the Mobile Sense App?	0	19 (82.6%)	4 (17.4%)
**3**	Does the collection of GPS data contribute to your sense of surveillance?	4 (17.4%)	12 (52.3%)	7 (30.3%)
**4**	Do you think it has been a burden or taken a lot of time to participate in the study?	1 (4.3%)	22 (95.7%)	0 (0%)

Table 3 shows that participants generally found the Mobile Sense App secure and not burdensome. Most (95.7%) did not find participation time-consuming, and none felt insecure about confidentiality. Furthermore, over half did not associate GPS tracking with surveillance. However, the majority (73.9%) were unwilling to share similar data in other contexts.

Moreover, the open-ended feedback provided additional insights into the app’s usability from the end-user perspective. Several recurring themes were identified, such as ease of use, need for reminders, login clarity, flexibility, and customization. In particular, participants found the app intuitive and quick to use: *“I appreciate that it's easy to use, that it goes fast to take and upload pictures, and finally I thought it was a good and short weekly questionnaire.”*

The most frequent suggestion was to strengthen the reminder function for logging foods and drinks: *“It was also easy to forget to take a photo of your food, so it would have been good to get a reminder around lunch every day.”* Furthermore, a few participants reported difficulties with the login process, mainly related to initial password instructions. Some participants suggested greater flexibility, such as customizable reminder times or the ability to copy repeated meals: *“It would also be good if you could set the time when the reminder comes… or copy previous days’ lunches if you buy the same thing every day.”*

Based on these insights, refinements were made to improve the reminder function and clarify log in instructions. While not all suggestions were implemented, targeted adjustments were prioritized to enhance usability for the pilot phase.

### Characteristics of photographs based on photo annotation system

Table 4 summarizes the annotations of photographs submitted by participants during working hours, capturing meals, snacks, and drinks consumed.

**Table 4.** Annotation of meal, snack, and drink photographs submitted by participants.

Photograph overview	Total (n=1178)
**Type of photograph**	**n (%)**
Relevant	1060 (90%)
Irrelevant	118 (10%)
**Relevant photograph**	
Meals	689 (70%)
Drinks	300 (30%)
**Meals and snacks**	
Full plate	674 (98%)
Empty plate	15 (2%)
**Drinks**	
Full glass	296 (99%)
Empty glass	4 (1%)

Out of the n=1178 photographs submitted, 90% (n=1060) were deemed relevant, while the remaining 10% (n=118) were excluded, as they depicted non-food content, such as streets or office scenes. Among the relevant photographs, 70% were meals and 30% drinks. Nearly all meal photographs depicted full plates (98%), with only 2% showing empty plates. Similarly, 99% of drink photographs displayed full glasses.

Table 5 presents the analysis of n=674 photographs of meals and snacks. Of these, 46% were home-made and 49% store-bought, with few originating from restaurants (4%), cafés (1%), and fast-food outlets (<1%). Overall, 40% contained at least one UPF item. Among the n=271 UPF items identified, sweet snacks were most common (56%), followed by refined grains (20%), ready-to-eat meals (15%), processed meats (10%), and sauces (7%). Plant-based products (5%), savoury snacks (1%), and fast food (0%) were infrequent.

**Table 5.** Characteristics of meal and snack photographs submitted by participants using the photo annotation system.

Meals and snacks	Total (n=674)
**Source of meal**	**n (%)**
Home-made	313 (46%)
Store-bought	329 (49%)
Café	6 (1%)
Fast food	1 (0%)
Restaurant	25 (4%)
**UPF prevalence**	
UPF	271 (40%)
Non-UPF	403 (60%)
**Type of UPF**	
Sweet snack	159 (56%)
Savory snack	4 (1%)
Junk food/Fast food	1 (0%)
Plant-based product	13 (5%)
Processed meat	27 (10%)
Ready to heat/eat meal	42 (15%)
Refined grains	54 (20%)
Sauce	18 (7%)

UPF, ultra-processed food.

Table 6 summarizes n=296 drink-related photographs. Most drinks were store-bought (83%), while 17% were home-made. The majority (87%) were unsweetened; 9% contained sugar, and 4% artificial sweeteners. Overall, 12% of drinks were classified as UPFs. Among the n=37 UPF drinks, sodas and flavored waters were the most prevalent (51%), followed by plant-based drinks (19%), and smaller proportions of juices (11%).

**Table 6.** Characteristics of drinks photographs submitted by participants using the photo annotation system.

Drinks	Total (n=296)
**Source of drink**	**n (%)**
Home-made	49 (17%)
Store-bought	247 (83%)
**Type of sweetener**	
Sugar sweetened	26 (9%)
Artificially sweetened	11 (4%)
Not sweetened	259 (87%)
**UPF prevalence**	
UPF	37 (12%)
Non-UPF	259 (88%)
**Type of UPF**	
Energy drink	2 (5%)
Protein drink	3 (8%)
Milkshake	2 (5%)
Soda/Flavored water	19 (51%)
Plant-based drink	7 (19%)
Juice	4 (11%)

UPF, ultra-processed food.

### Prevalence of UPFs in home-made vs. store-bought meals

**Figure 6.**
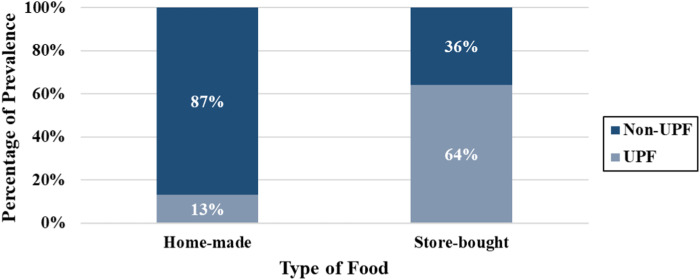
Prevalence of ultra-processed food items in home-made vs. store-bought meals and snacks based on image annotation system.

Figure 6 illustrates the prevalence of UPFs in home-made versus store-bought meals and snacks. Among n=313 home-made items, 87% were non-UPF and 13% contained UPFs. In contrast, of n=361 store-bought items, 64% were UPFs and 36% non-UPF. The difference in UPF prevalence between home-made and store-bought items was statistically significant (χ^2^ (1)=178.63, p<.001), indicating a substantially higher likelihood of UPF content in store-bought meals.

### Geospatial heatmap analysis of food purchasing

**Figure 7.**
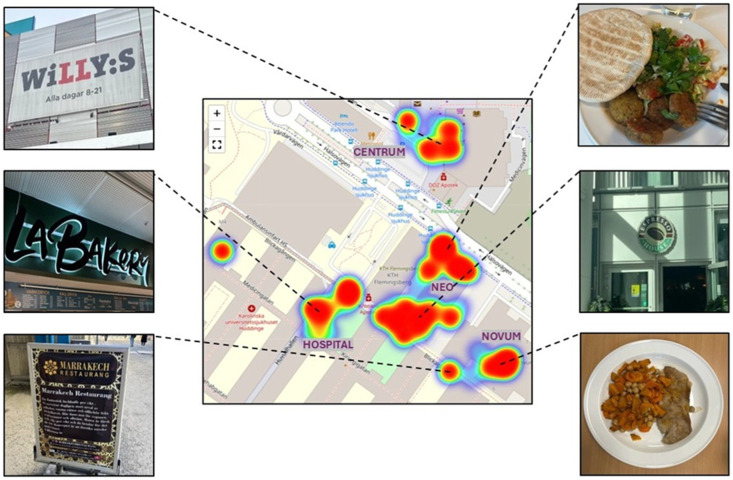
Heatmap of Global Position System (GPS) data, showing where participants purchased and consumed food around the Karolinska Institute (KI) South Campus. The depicted pictures are examples of the locally contributed data.

Figure 7 presents a heatmap of the KI South campus, illustrating the distribution of food-related behaviors during work hours, based on geo-tagged photographs submitted by participants. Activity intensity is indicated by color gradients, with warmer tones (red and orange) representing higher frequencies of food-related events, and cooler tones (blue and green) indicating lower activity. Clusters of activity are observed around Karolinska University Hospital and the attached research buildings. Specifically, all the submitted photographs fell within a 556 meter radius around the Neo Research building, indicating that all food-related behaviors occurred in these areas.

## Discussion

This study evaluated the feasibility and usability of the Mobile Sense App for capturing eating behaviors and food environment exposure in a real-world university setting. Overall, the app proved feasible and usable, as demonstrated by the SUS score (mean 75.2 ± 12.2), placing it above the established usability benchmark for digital health applications (range 68-70).^28^ The adherence patterns visualized in Figure 4 further support the app’s feasibility, illustrating consistent user engagement over the observation period, with visible peaks of high contribution in the initial days followed by a gradual decline toward the end of data collection. From a data quality perspective, photographs collected during participants’ working hours predominantly consisted of relevant images depicting meals, snacks, and drinks. Only a small proportion of photographs were excluded for being unrelated to the study focus (e.g., non-food items).

Furthermore, usability questions and open-ended feedback highlighted desired features, such as reminders and greater flexibility that would enhance usability and engagement. In particular, open-ended feedback revealed consistent themes around the need for reminders, clarity in user interface (UI) instructions, and greater flexibility in meal and annotation options. Participants generally received the app as intuitive and easy to use but emphasized that engagement depended on timely prompts and clear onboarding instructions. These kinds of refinements are often cited in mobile health usability research as essential for improving engagement and reducing attrition over time.^29^ At the same time, studies show that usability is strongly tied to both technical performance (ease of use, clarity, speed) and user experience (flexibility of app to personal preferences, interface clarity).^29,30^ Collectively, these findings suggest that the Mobile Sense App is a practical and acceptable tool for short-term dietary assessment in workplace settings, with clear opportunities for further optimization.

The preference for home-made meals aligns with previous studies suggesting that individuals with higher health literacy, or those working in health-promoting environments, are more likely to choose home-made foods and limit UPF consumption.^31^ Specifically, participants reported eating home-made meals 2-3 times per week, while an even larger number indicated doing so 4-5 times per week. Additionally, participants reported low consumption of unhealthy food and drink options, including energy drinks, sweetened and artificially sweetened beverages, and fast food (Figure 3). Nevertheless, the notable proportion of store-bought meals highlights the persistent influence of convenience and accessibility in food choices. Prior research shows that workplace eating behaviors are often shaped by time pressure, food availability, and the perceived convenience of ready-to-eat items.^32,33^ Furthermore, a markedly higher prevalence of UPFs was observed in store-bought meals and snacks compared with home-made ones, supporting previous evidence linking convenient and ready-to-eat foods to higher UPF content and poorer overall dietary quality.^10,34^

Spatial analyses further revealed that all food-related activities occurred within 556 meter radius to the workplace and lecture halls, particularly near areas dense with restaurants, cafés, and food outlets. This pattern reinforces the notion that proximity and convenience are primary drivers of food purchasing behavior, a finding consistent with existing literature on environmental determinants of dietary choices.^35^

From a public health perspective, these findings emphasize the need to improve the accessibility and appeal of minimally processed, nutritious foods across universities and broader occupational environments. This is particularly relevant given the growing body of evidence linking higher consumption of UPFs to adverse health outcomes, including obesity, type 2 diabetes, chronic kidney disease, and increased mortality.^7,36,37^ Creating supportive food environments, through healthier procurement policies, transparent labeling, and increased availability of minimally processed options can help reduce reliance on UPFs and promote more sustainable eating behaviors.^38^ This aligns with global frameworks such as the EAT Lancet Commission’s recommendations on sustainable diets,^38^ as well as Swedish national priorities that position food environments as key determinants of healthier diets.^39^

Overall, the convergence of dietary and spatial data in this study demonstrates that the food environment is a determinant of dietary quality, linking accessibility, convenience, and occupational infrastructure to everyday food choices. Addressing these environmental drivers is therefore essential for fostering healthier and more sustainable eating patterns in occupational settings.

### Strengths and limitations

This study presents several methodological and technological strengths that enhance its applicability. To our knowledge, it is the first investigation to employ a mobile app for assessing both eating behaviors and food environment in a university setting.

The integration of geolocation and timestamp data allowed for a detailed mapping of food-purchasing ‘hotspots’, providing novel insights into the spatial dynamics of food behaviors in workplace and university contexts.^40^ Moreover, the inclusion of engagement metrics, such as a photograph submission heatmap, and the combination of quantitative usability scores with qualitative feedback, provided a multidimensional understanding of user experience.

Nevertheless, several limitations should be acknowledged. The sample, composed primarily of university employees and students within a health-oriented academic environment comprised predominantly by females, reflects a specific organizational context and may limit the generalizability of the results to other occupational settings. However, this context also allowed for a more controlled examination of eating behaviors within a defined food environment. The relatively short two-week data collection period provided only a snapshot of participants’ eating behaviors and may not reflect long-term variations in dietary patterns and engagement with the food environment. This time frame was selected to balance participant burden with the collection of detailed, real-time data and was considered sufficient to capture recurring, routine eating behaviors within individuals. Although the use of photographic food logging reduced recall bias, participants’ awareness of being monitored may have introduced reactivity or socially desirable reporting, potentially influencing their food choices.^41^ In addition, while standardized annotation procedures minimize misclassification, photographs alone cannot capture hidden ingredients, which may introduce errors in NOVA-based UPF classification.^42^

The data collection approach also raised potential privacy concerns. To address this, each participant was assigned a unique trial ID, and all data were stored without personal identifiers (e.g., names, dates of birth). The main server applied automated face-censoring to unintended images to ensure anonymity (Supplementary Figure 3).

## Conclusion

The demonstrated feasibility and usability of the Mobile Sense App support its potential for large-scale deployment in diverse populations and work environments, particularly where continuous, real-time dietary and food environment analytics monitoring are needed. Future research should focus on optimizing app functionalities, such as customizable reminders, automated food recognition, and adaptive interfaces that respond to user behavior over time to sustain engagement. Additionally, it should extend this approach to more diverse populations to further validate the methodology and assess the stability of the observed behaviors. Integrating machine learning or artificial intelligence-based annotations could substantially enhance scalability by reducing manual workload, while improving the accuracy of UPF classification. By mapping food-purchasing ‘hotspots’ and quantifying UPF exposure, this approach can advance evidence-based strategies to promote healthier eating behaviors and food environments in occupational settings.

## Supplemental material

Supplemental material - Methodology development for analyzing eating behavior, diet quality, and food environment in occupational settings Supplemental material for Methodology development for analyzing eating behavior, diet quality, and food environment in occupational settings by Valeria Cecchini, Negar Zand Miralvand, Vasileios Papapanagiotou, Carla Maria Avesani, and Ioannis Ioakeimidis in Digital Health.

## Data Availability

Data are available upon reasonable request.*
