# Supplementary material for: Methodology development for analyzing eating behavior, diet quality, and food environment in occupational settings
Source: Digit Health. 2026 Jul 16;12:20552076261467799. doi: 10.1177/20552076261467799 (PMC13376500; doi:10.1177/20552076261467799)
Supplement: Supplemental material - Methodology development for analyzing eating behavior, diet quality, and food environment in occupational settings [file sj-pdf-1-dhj-10.1177_20552076261467799.pdf]

Supplementary material

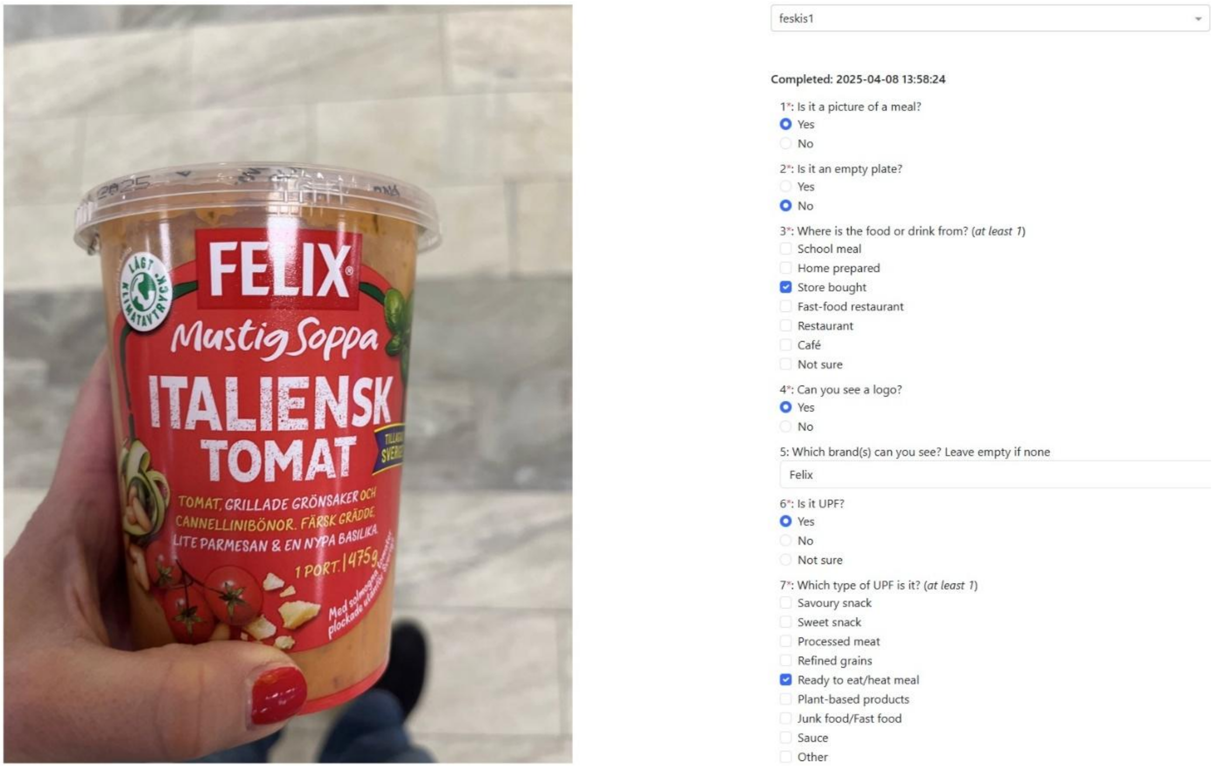

Supplementary Figure 1. Screenshot of meal photograph annotating procedure in the image annotation system.

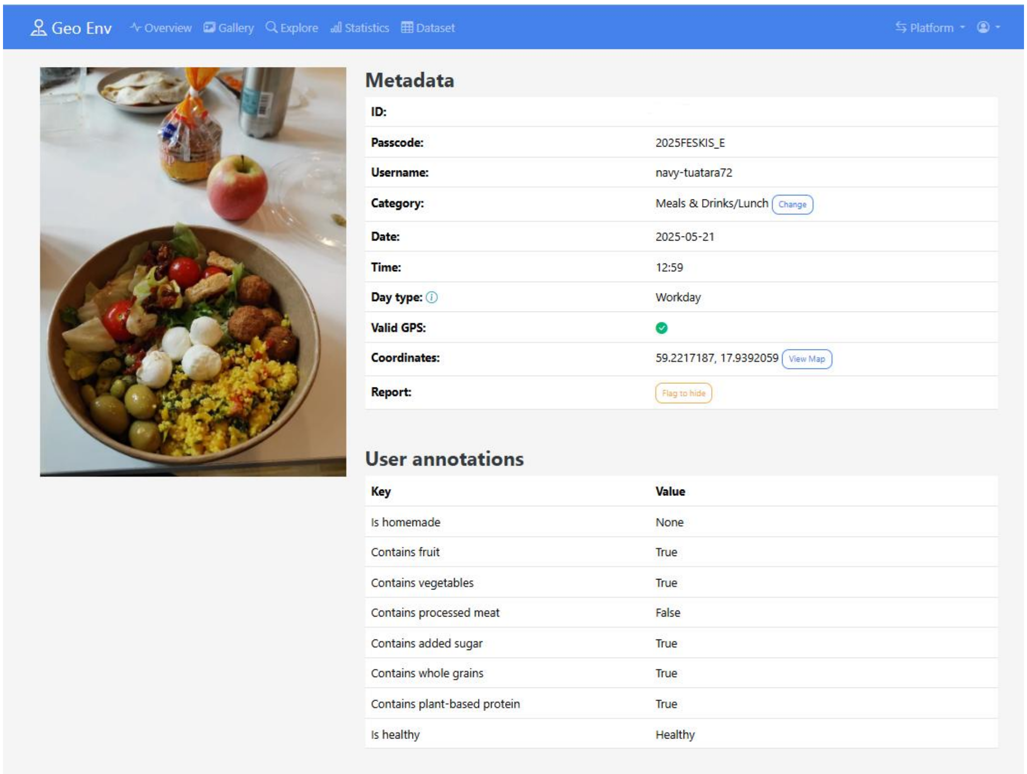

Supplementary Figure 2. Example of meal picture metadata in the Arbisense Portal.

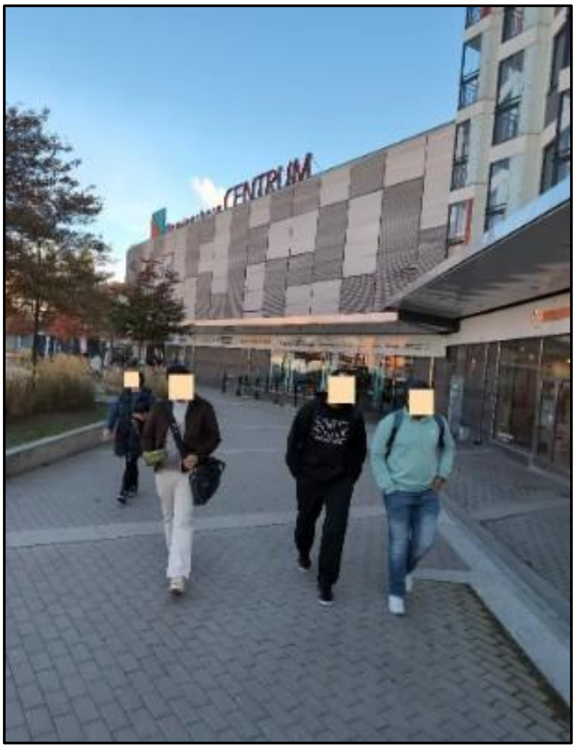

Supplementary Figure 3. Automatic face-censoring technology in the main portal.
